# 89. First-in-human study of a novel half-life extended monoclonal antibody (GB-0669) against SARS-CoV2 and related sarbecoviruses

**DOI:** 10.1093/ofid/ofae631.026

**Published:** 2025-01-29

**Authors:** Dinesh De Alwis, Francesco Borriello, Eric Carlin, Rounak Nassirpour, Stephanie Straley, Anna Allen, Andrew W Robertson, Iñaki F Troconiz, Akber Safder, Daria Hazuda, Lovely Goyal, Gavin CKW Koh, Nicholas Robertson, Alexandra Snyder

**Affiliations:** Generate Biomedicines, New York, NY; Generate Biomedicines, New York, NY; Generate Biomedicines, New York, NY; Generate Biomedicines, New York, NY; Generate Biomedicines, New York, NY; Generate Biomedicines, New York, NY; Generate Biomedicines, New York, NY; University of Navarra, Pamplona, Navarra, Spain; PPD part of Thermofisher, Orlando, Florida; Generate Biomedicines, New York, NY; Generate Biomedicines, New York, NY; Generate:Biomedicines, Somerville, Massachusetts; Generate Biomedicines, New York, NY; Generate Biomedicines, New York, NY

## Abstract

**Background:**

GB-0669 is a novel half-life extended monoclonal antibody for the prophylaxis of SARS-CoV2 infection and is one of the first biologics designed using artificial intelligence/machine learning to reach clinical development and the first against SARS-CoV2. GB-0669 targets the previously undruggable S2 domain of the SARS-CoV2 spike protein, which contains the fusion peptide and stem-helix peptides, and was selected because the S2 domain is conserved across all SARS-CoV2 variants to-date. The S2 domain is not immunodominant and is therefore not subject to selective pressure from natural infection or vaccine induced immunity. This suggesting GB-0669 will maintain its activity, unlike monoclonal antibodies directed against the SARS-CoV2 receptor-binding domain. GB-0669 is also able to neutralize other sarbecoviruses, such as SARS-CoV2 and WIV1 (Table).

**Table:**
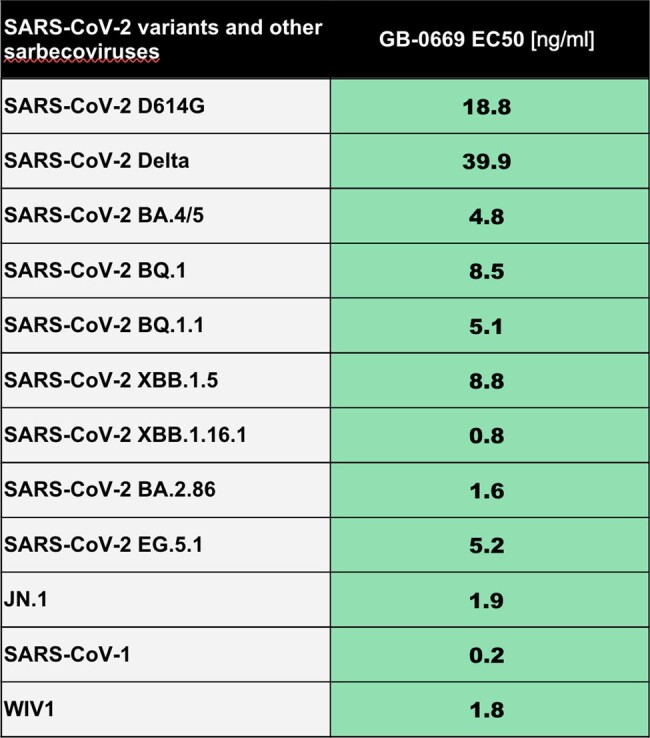
GB-0669 EC50 against SARS-CoV2 variants and other sarbecoviruses in a VSV pseudoneutralisation assay

**Methods:**

In this single-ascending dose study, healthy volunteers aged 18–55 years were administered either GB-0669 or placebo intravenously. There were five sequential dosing cohorts 100 mg, 300 mg, 600 mg, 1200 mg, 2400 mg and each cohort was randomized to receive either GB-0669 or placebo in ratios of 3:3, 3:3, 10:3, 10:3, 10:3 respectively. Total follow-up will be 43 weeks; although dosing is now complete, follow-up is ongoing.

**Figure:**
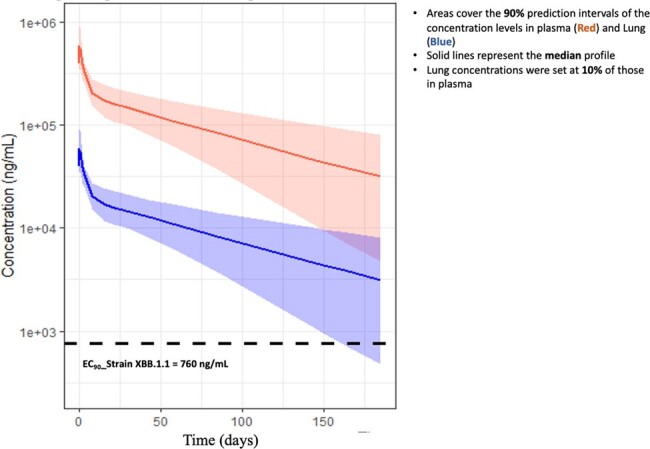
Simulated GB-0669 drug concentrations over 43 weeks (plasma and lung) following a single dose of 1200 mg

**Results:**

No dose-limiting toxicity has been observed to date and all treatment-related adverse reactions observed were mild (grade 1 or 2) at all doses. Preliminary pharmacokinetic data shows dose-proportionality up to 2400 mg the highest dose tested, with a half-life of approximately 70 days. An exploratory analysis of live virus neutralization shows a clear dose response and separation from placebo at 600 and 1200 mg. Limited anti-drug antibodies were observed in a few participants, but had no observable impact on PK or viral neutralization titers. Simulated PK of GB-0669 confirmed with observed data (assuming 10% lung penetration) are at levels anticipated to protect against COVID-19 for at least 150 days (Figure).

**Conclusion:**

The preliminary data support the hypothesis that GB-0669 will be able to provide a six-month duration of prophylactic efficacy against currently circulating viral variants when administered as part of a combination.

**Disclosures:**

**Dinesh De Alwis, PhD**, Generate Biomedicines: Stocks/Bonds (Private Company) **Iñaki F. Troconiz, Pharmacy**, Generate Biomedicines: Advisor/Consultant **Daria Hazuda, PhD**, Generate Biomedicines: Stocks/Bonds (Private Company) **Gavin CKW Koh, PhD**, AstraZeneca: Stocks/Bonds (Public Company)|Generate:Biomedicines: Stocks/Bonds (Private Company)|GlaxoSmithKline: Stocks/Bonds (Public Company) **Alexandra Snyder, MD**, Generate Biomedicines: employee

